# Monitoring of Human Cytomegalovirus and Virus-Specific T-Cell Response in Young Patients Receiving Allogeneic Hematopoietic Stem Cell Transplantation

**DOI:** 10.1371/journal.pone.0041648

**Published:** 2012-07-25

**Authors:** Daniele Lilleri, Giuseppe Gerna, Paola Zelini, Antonella Chiesa, Vanina Rognoni, Angela Mastronuzzi, Giovanna Giorgiani, Marco Zecca, Franco Locatelli

**Affiliations:** 1 Laboratori Sperimentali di Ricerca, Area Trapiantologica, Fondazione IRCCS Policlinico San Matteo, Pavia, Italy; 2 Struttura Semplice Virologia Molecolare, Struttura Complessa Virologia e Microbiologia, Fondazione IRCCS Policlinico San Matteo, Pavia, Italy; 3 Dipartimento di Oncoematologia Pediatrica, IRCCS Ospedale Pediatrico Bambino Gesù, Roma, Italy; 4 Struttura Complessa di Ematologia ed Oncologia Pediatrica, Fondazione IRCCS Policlinico San Matteo, Pavia, Italy; 5 Università di Pavia, Pavia, Italy; Hospital Infantil Universitario Niño Jesús, Spain

## Abstract

In allogeneic hematopoietic stem-cell transplantation (HSCT) recipients, outcome of human cytomegalovirus (HCMV) infection results from balance between viral load/replication and pathogen-specific T-cell response. Using a cut-off of 30,000 HCMV DNA copies/ml blood for pre-emptive therapy and cut-offs of 1 and 3 virus-specific CD4^+^ and CD8^+^ T cells/µl blood for T-cell protection, we conducted in 131 young patients a prospective 3-year study aimed at verifying whether achievement of such immunological cut-offs protects from HCMV disease. In the first three months after transplantation, 55/89 (62%) HCMV-seropositive patients had infection and 36/55 (65%) were treated pre-emptively, whereas only 7/42 (17%) HCMV-seronegative patients developed infection and 3/7 (43%) were treated. After 12 months, 76 HCMV-seropositive and 9 HCMV-seronegative patients (cumulative incidence: 90% and 21%, respectively) displayed protective HCMV-specific immunity. Eighty of these 85 (95%) patients showed spontaneous control of HCMV infection without additional treatment. Five patients after reaching protective T-cell levels needed pre-emptive therapy, because they developed graft-versus-host disease (GvHD). HSCT recipients reconstituting protective levels of HCMV-specific T-cells in the absence of GvHD are no longer at risk for HCMV disease, at least within 3 years after transplantation. The decision to treat HCMV infection in young HSCT recipients may be taken by combining virological and immunological findings.

## Introduction

Human cytomegalovirus (HCMV) still represents the most important viral infection in allogeneic hematopoietic stem cell transplantation (HSCT) recipients [1]. Following the identification of the most sensitive diagnostic procedures for detection and quantification of HCMV in blood [2]–[6], prevention of HCMV infection/disease was achieved by adoption of either universal prophylaxis (i.e. treatment of all HSCT recipients with anti-HCMV drugs starting from the day of transplantation/engraftment through 3–6 months thereafter) or pre-emptive therapy (i.e. starting treatment upon detection of HCMV in blood at predetermined cut-off levels until its confirmed disappearance from blood) [7]–[9]. However, with either approach, a minority of patients display recurrent episodes of HCMV infection, following discontinuation of antiviral treatment either administered prophylactically (late disease) or pre-emptively (episodes of HCMV reactivation). The variability in the efficacy of antiviral treatment in different patients was related to differences in the immune reconstitution process (in HCMV-seropositive patients) or to the development of the HCMV-specific T-cell immune response (in HCMV-seronegative patients) [10], [11].

Although results reported on this subject have been somewhat controversial, also due to use of different methodologies for evaluating virus-specific immunity (MHC-peptide tetramer technology or intracellular cytokine staining following stimulation with peptide pools or HCMV-infected cell lysate), the conclusion of some authors was that HCMV-specific CD8^+^ T-cells were sufficient to provide permanent protection against HCMV reactivation [12], [13]. Other reports found that HCMV-specific CD4^+^ T-cells were required to confer protection [14], [15]. Our recently introduced methodology for assessment of specific immunity, based on T-cell stimulation by autologous, monocyte-derived, HCMV-infected dendritic cells [16], has been shown to provide a comprehensive evaluation of both CD4^+^ and CD8^+^ T-cell response in immunocompromised hosts [17].

Since a long-term follow-up study, monitoring in parallel HCMV load and T-cell immune response, has not been conducted so far, in this study, we measured in parallel HCMV DNA load in blood and HCMV-specific CD4^+^ and CD8^+^ T-cells producing both interferon-γ (IFN-γ) and interleukin-2 (IL-2) in 131 young HSCT recipients. We aimed at verifying whether achievement of previously established protective levels of T-cell response were able to prevent HCMV reactivation episodes in the absence of other interfering immunosuppressive factors or events, such as graft-versus-host disease (GvHD) occurrence.

## Materials and Methods

### Patients and Study Design

From January 2007 through January 2010, a total of 131 young patients receiving allogeneic HSCT were enrolled in this study; patient characteristics are reported in Table 1. Inclusion criteria were: i) patients receiving any type of allogeneic HSCT; ii) donor, recipient or both having serological evidence of past HCMV infection; iii) patients or their parents having provided informed written consent in accordance with the declaration of Helsinki.

**Table 1:** Characteristics of the 131 patients analyzed.

Characteristics		Patient no. (%)
Gender (M/F)		42/89
Median age at transplantation (range)		8 (1–23) years
Underlying disease	malignant	98 (75)
	non-malignant	33 (25)
Stem cell source	bone marrow	78 (60)
	peripheral blood	42 (32)
	cord blood	11 (8)
Donor type	sibling	45 (34)
	unrelated	57 (44)
	haploidentical relative	29 (22)
Donor/recipient HCMV serostatus	D^+^/R^+^	51 (39)
	D^−/^R^+^	38 (29)
	D^+^/R^−^	42 (32)
Conditioning regimen	TBI-based	61 (47)
	chemotherapy-based	70 (53)
GvHD prophylaxis	CS-A	22 (17)
	MTX	1 (1)
	CS-A + MTX	69 (53)
	CS-A + steroid	11 (8)
	T-cell depletion	28 (21)
Administration of ALG	Yes	86 (66)
	No	45 (34)
GvHD	Acute (grade II–IV)	42 (32)
	Chronic	24 (18)
Graft failure,		
-median days (range) after transplantation:	442 (49–528)	3 (2)
Disease relapse,		
-median days (range) after transplantation:	247 (35–643)	35 (36)*
Transplantation-related death,		
-median days (range) after transplantation:	216 (57–869)	7 (5)

TBI: total body irradiation; GvHD: graft *vs* host disease; CS-A: cyclosporine-A; MTX:methotrexate; ALG: anti-lymphocyte globulin methotrexate; ALG: anti-lymphocyte globulin.

* Among patients with malignant disease.

The immune response was considered protective when it could control infection in at least 95% cases. On the basis of a previous study [17], we chose levels of at least 1 HCMV-specific CD4^+^ and 3 CD8^+^ T cells/µL blood (in the absence of anti-GvHD treatment) as immunological cutoffs. In this case, the proportion of patients developing HCMV disease or reaching 30,000 HCMV DNA copies/µL blood (the cutoff currently used for initiating preemptive therapy) in the presence of at least 1 HCMV-specific CD4^+^ and 3 CD8^+^ T cells/µL blood should be less than 5%. Assuming a study power of ≥0.80 and using a binomial distribution model to calculate the 95% confidence interval for the failure rate, the upper limit of this interval would be ≤5% if no more than 3 out of 130 patients develop HCMV disease or reach the cutoff for pre-emptive therapy after immune recovery.

The study protocol was approved by the Ethics Committee of Fondazione Policlinico San Matteo on November 13, 2006 (procedure no. P-20060028979).

### Virologic Monitoring

HCMV infection was diagnosed following HCMV detection in blood in the absence of clinical symptoms or organ function abnormalities, while HCMV disease was defined as either systemic or local, when HCMV infection was associated with clinical symptoms and/or organ function abnormalities [18].

Patients were monitored for HCMV infection in blood by determination of DNA level in blood (DNAemia) bi-weekly from day 0 until discharge from the hospital, and then once weekly for the first three months [19]. Subsequently, patients were monitored for HCMV upon control medical visits or in the presence of clinical symptoms suggestive of HCMV infection. In case of patients requiring immunosuppressive therapy for GvHD, weekly monitoring of HCMV was resumed. In any case, after HCMV DNA was detected in blood, bi-weekly monitoring of DNAemia and viremia [20] was performed. Pre-transplantation donor/recipient HCMV serostatus was determined according to previously reported methods [21].

No patient received HCMV prophylaxis. Pre-emptive therapy was administered to patients with an HCMV DNAemia ≥30,000 copies/ml whole blood. This cut-off was elevated with respect to previous studies [17], [21], [22] from 10,000 to 30,000 copies/ml whole blood since symptomatic HCMV recipients observed along the years never showed DNAemia lower than 70,000 copies/ml. Such an increase in HCMV DNA cut-off was introduced to minimize, during immune reconstitution, pre-emptive treatment in those patients who have an HCMV viral load still in the range of asymptomatic infection. Antiviral preemptive therapy consisted of administration of intravenous ganciclovir (10 mg/kg/day), replaced by foscarnet (PFA, 180 mg/Kg/day) in case of neutropenia (≤500 neutrophils/µl) or increasing DNAemia despite therapy. PFA was also given to patients receiving either cord blood transplantation (CBT) or T-cell depleted HSCT in case of HCMV detection in blood before engraftment. Antiviral treatment was stopped following two consecutive negative blood controls.

### Management of Patients for HSCT

All patients received fully myeloablative preparative regimen. GvHD prophylaxis consisted of cyclosporine-A (Cs-A) either alone or associated with short-term methotrexate (MTX) for patients receiving an HLA-identical sibling allograft. Patients transplanted from an unrelated donor were given anti-lymphocyte globulin (ALG) on days -4, -3 and -2 before transplantation in addition to Cs-A and short-term MTX (this latter drug being substituted by steroids in patients receiving CBT). ALG administration and T-cell depletion of the graft were used for patients given HSCT from an HLA-haploidentical relative. Acute GvHD was initially treated with steroids, whereas patients with steroid-resistant disease received extracorporeal photochemotherapy [23], mycophenolate mofetil (MMF) and mesenchymal stromal cells.

### Immunologic Follow-up

Absolute CD3^+^CD4^+^ and CD3^+^CD8^+^ T-cell counts were determined on peripheral blood by direct immunofluorescence flow-cytometry (Beckman Coulter Inc, Fullertone, CA, USA). The frequency of HCMV-specific CD4^+^ and CD8^+^ T-cells producing IFN-γ and IL-2 was determined by cytokine flow-cytometry, following *in vitro* stimulation with autologous monocyte-derived, HCMV-infected, dendritic cells, as reported [16]. The absolute number of HCMV-specific CD4^+^ and CD8^+^ T-cells producing IFN-γ and IL-2 was calculated by multiplying the percentage of HCMV-specific T-cells by the relevant absolute number of CD4^+^ and CD8^+^ T-cells.

Using this methodology, HSCT recipients were considered HCMV-immune when reaching levels of both HCMV-specific CD4+ and CD8+ T-cell counts greater than 1 and 3 cells/µl whole blood, respectively, as previously reported [17], [21]. Immunological assays were performed monthly until day 180 after transplantation, then every 3 months until detection of HCMV-specific CD4^+^ and CD8^+^ T cells. Clinical/immunological/virological surveillance was continued for a minimum of one year unless other events (such as transplant rejection or relapse of the underlying disease) occurred, in which cases immunological follow-up was stopped. In HCMV-seronegative patients showing neither HCMV infection nor HCMV-specific T-cell response 6 months after transplantation, immunological follow-up was stopped, due to the negligible chance that they should develop either infection or immunity thereafter, as previously observed [17].

### Statistical Analysis

Data were analyzed as of 1 May 2011, after a median follow-up of 966 (49–1559) days. The probability of developing HCMV infection and HCMV-specific immune response, the rates of transplantation-related mortality (TRM) and GvHD (acute and chronic) were expressed as cumulative incidence, taking into account the appropriate competing risks. Event-free survival (EFS) and overall survival (OS) were calculated by the Kaplan-Meyer method. Differences between groups were calculated by the log-rank test or the Gray test, as appropriate. P values lower than .05 were considered statistically significant. Spearman correlation was calculated between time to detection of HCMV-specific T-cell immunity and time to clearance of virus from blood (confirmed absence of DNAemia). A Cox proportional hazard regression model was used to analyze in multivariate analysis factors potentially associated with delay in HCMV-specific immune reconstitution.

## Results

### Development of HCMV Infection

**Figure 1:**
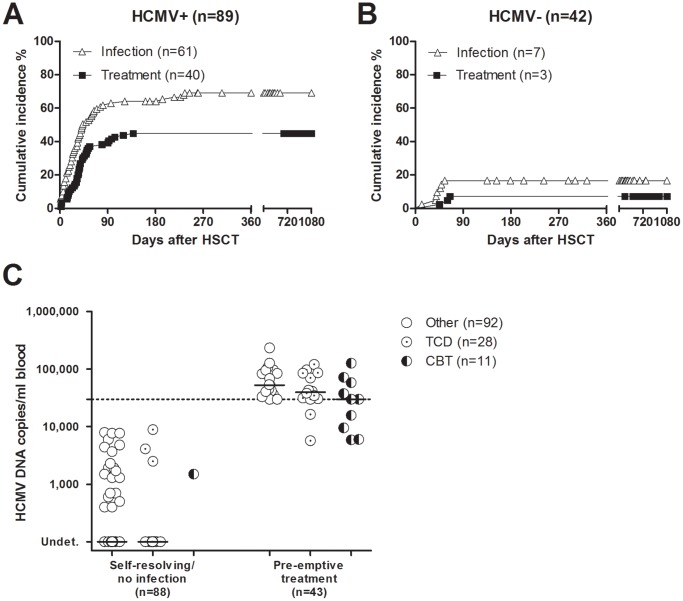
Cumulative incidence of HCMV infection in 131 young patients receiving HSC transplantation. (A) HCMV-seropositive patients. (B) HCMV-seronegative patients. (C) HCMV viral load in 88 patients with self-resolving or no HCMV infection, and in 43 patients requiring antiviral treatment. Among patients receiving T-cell depleted transplantation (TCD), 14/28 were included in the pre-emptive treatment group. Similarly, 10/11 patients receiving cord blood transplantation (CBT) were included in the pre-emptive treatment group.

Among the 89 HCMV-seropositive patients, HCMV infection occurred in 55 patients (62%) in the course of the first 3 months after transplantation, and in 6 additional patients between 4 and 8 months after transplant (Fig. 1A). Thus, the 1-year cumulative incidence of HCMV infection was 69% (95% confidence interval -CI-: 63–77%) with a median interval of 27 days (range: 0–215) between transplantation and HCMV infection. Pre-emptive therapy was administered to 36 of the 55 (65%) patients with HCMV infection within the first 3 months. Six of them (4 receiving unrelated donor CBT and 2 receiving a T-cell depleted HSCT from an HLA-haploidentical relative) were given PFA, before reaching the cut-off of 30,000 DNA copies/ml blood, because of detection of increasing HCMV DNA levels in blood prior to engraftment (Fig. 1C). Four additional patients reached the cut-off for pre-emptive therapy between 97 and 138 days after transplantation. One patient, following a first course of pre-emptive therapy and a subsequent HCMV relapse episode in blood, developed HCMV gastritis (day +139) after spontaneous disappearance of virus from blood. This was the only patients of the whole cohort developing HCMV disease.

In the group of 42 HCMV-seronegative patients, 7 (17%) developed HCMV infection, and 3 (7%) received pre-emptive therapy (Fig. 1B). In this subgroup, the 1-year cumulative incidence of HCMV infection was 17 (95% CI: 3–43), with a median interval of 41 days (range: 12–55) between transplantation and HCMV infection.

Concerning the distribution of patients requiring pre-emptive therapy, 19/92 (21%) patients receiving unmanipulated bone marrow or peripheral blood HSCT, 14/28 (50%) patients receiving T-cell depleted transplantation and 10/11 (91%) patients receiving CBT were given antiviral treatment.

The median HCMV-DNAemia for the 43 treated patients (40 HCMV-seropositive and 3 HCMV-seronegative) was 42,900 copies/ml (range 5,700–233,900, Fig. 1C). In the 88 patients (49 HCMV-seropositive and 39 HCMV-seronegative) not requiring antiviral treatment due to self-resolving infection or absence of infection, median viral load level was 0 (range 0–8,900) DNA copies/ml blood. Antiviral treatment was given for an overall median time of 27 days (range 10–99), and was able to clear virus from blood in all patients but two, who died due to GvHD before virus clearance.

### Outcome of Transplantation

**Figure 2:**
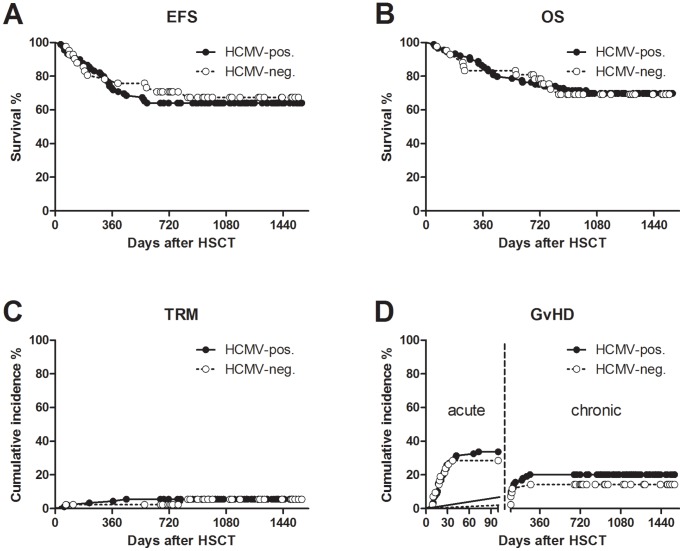
Probability of survival, transplant-related mortality and GvHD in the HSCT studied population. (A) event-free survival (EFS), (B) overall survival (OS): no significant difference was found by the log-rank test. (C) Transplantation–related mortality (TRM), and (D) acute and chronic GvHD were expressed as cumulative incidence, taking into account the appropriate competing risks: no difference was found by the Gray test. HCMV-seropositive and HCMV-seronegative young HSCT recipients are reported separately.

For the whole cohort of patients, the 3-year EFS and OS probabilities were 66% (95% CI: 59–75%) and 71% (95% CI: 64–80%), respectively. Seven patients died for transplantation-related causes, and the 3-year cumulative incidence of TRM was 6% (95% CI: 0–26). In addition, 42 and 24 patients experienced grade II-IV acute or chronic GvHD; the cumulative incidence of grade II-IV acute GvHD at 100 days and that of chronic GvHD at 3 years post-transplant were 32% (95% CI: 21–44) and 18% (95% CI: 5–29%), respectively. Overall, 47 patients (36%) experienced acute and/or chronic GvHD, all within one year after HSCT. No significant difference was found between HCMV-seropositive and HCMV-seronegative HSCT recipients for both TRM and GvHD cumulative incidences (see also Fig. 2).

### Development of HCMV-specific T-cell Immune Reconstitution

**Figure 3:**
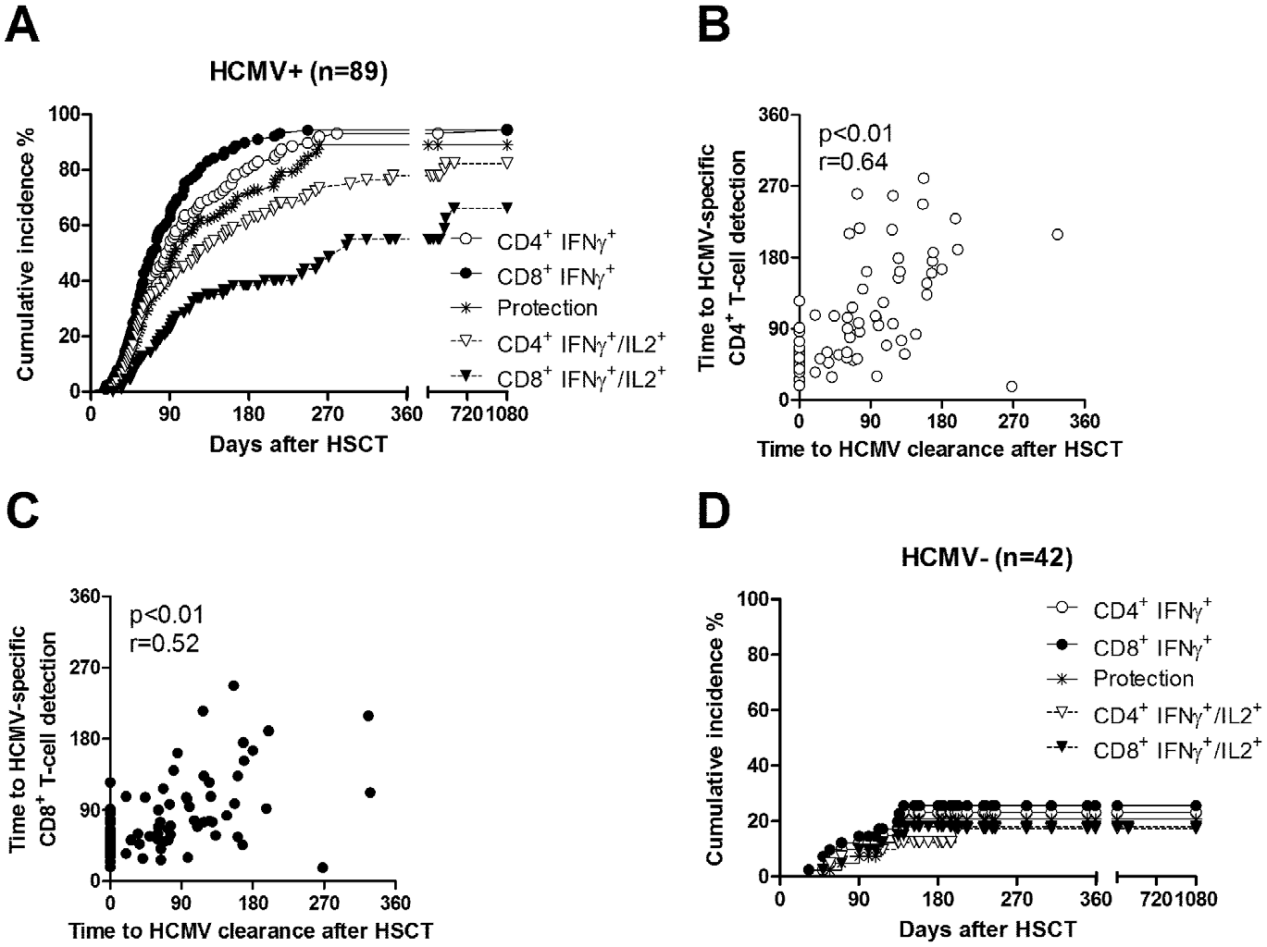
Cumulative incidence of IFN-γ^+^ and IFN-γ^+^/IL-2^+^ CD4^+^ and CD8^+^ T-cell recovery in HSCT recipients. (A) HCMV-seropositive and (D) HCMV-seronegative young HSCT recipients. The cumulative curve indicating levels of protection during follow-up is also reported. Five (20%) of the 25 patients who developed HCMV-specific CD8+ T-cell response only a median time of 57 (16–340) days prior to appearance of CD4+ T-cells, had high DNAemia levels requiring antiviral treatment. The correlation (Spearman correlation test) between time to protection by HCMV-specific (B) CD4^+^ or (C) CD8^+^ T-cells and time to HCMV clearance from blood is shown. Within 12 months after transplantation, 95/131 patients developed specific T-cell immunity: 2 CD8+ only, and 93 both CD4 and CD8 T-cells. Of these 93, 85 developed specific immunity above the cutoff levels established for immune compromised patients (but 5 required antiviral treatment because of steroid therapy for GvHD), and 8 only levels above the cutoffs established for immune competent subjects (and were found to be also protected from reactivation).

In the 89 HCMV-seropositive patients, appearance of HCMV-specific IFN-γ^+^ CD8^+^ T-cells preceded that of IFN-γ^+^ CD4^+^ T-cells, the median time to detection of the 2 subsets being 69 *vs* 84 days, respectively (p = 0.02, Fig. 3A). Nine months after transplantation, all surviving patients had both HCMV-specific CD4^+^ and CD8^+^ T-cells. A single patient did not show CD4+ T-cells after more than 1 year of follow-up.

IFN-γ^+^/IL-2^+^ CD4^+^ and CD8^+^ T-cells were found to emerge later than IFN-γ^+^ T-cells. Indeed, their median time of detection was 116 days for CD4^+^ and 260 days for CD8^+^ IFN-γ^+^/IL-2^+^ T-cells, respectively (Fig. 3A).

Reconstitution of protective immunity (i.e. presence of at least 1 HCMV-specific CD4^+^ and 3 CD8^+^ T-cells/µl blood) was documented in 76 seropositive patients, the cumulative incidence being 90% (95% CI: 87–93%) at nine months. In details, protective levels were achieved at 3 months by 50/84 (60% of event-free surviving patients, i.e. alive in the absence of rejection or disease relapse), at 6 months by 65/83 (78%), and at 9 months by 76/78 (97%) patients (Fig. 3A). The curve of cumulative incidence of “protective” immunity closely overlapped that of HCMV-specific CD4^+^ T-cells (Fig. 3A). The correlation between time to HCMV clearance from blood and time to HCMV-specific CD4^+^ and CD8^+^ T-cell detection was statistically (although not biologically) significant (p<0.01) for both subsets, and slightly greater for CD4^+^ (r = 0.64) than CD8^+^ (r = 0.52) T-cells (Fig. 3B, C).

In the 42 HCMV-seronegative patients, 9 (21%) developed both CD4^+^ and CD8^+^ T-cell HCMV-specific immunity within 12 months after transplantation. However, of these, 8 patients reconstituted specific immunity within 5 months (Fig. 3D), and 1 patient one year after transplantation. In addition, one HCMV-seronegative patient developed HCMV-specific CD4^+^ and CD8^+^ T-cells below the “protective” threshold levels, one patient developed only CD8^+^ specific T-cell immunity, and 6 a transitory, short-lived specific T-cell immunity.

The following factors potentially influencing HCMV-specific T-cell reconstitution were examined: gender, age, donor serostatus, conditioning regimen (total body irradiation-based *vs* chemotherapy-based), stem cell source, T-cell depletion, ALG administration, GvHD (acute and chronic). Among these, the only factors independently predicting a delay in the process of immune reconstitution were T-cell depletion of the graft (p<0.01) and CBT (p = 0.03) (data not shown). Donor serostatus did not show a significant impact on the reconstitution of either IFN-γ^+^ or IFN-γ/IL-2^+^ CD4^+^ and CD8^+^ T-cells (data not shown).

### Control of HCMV Infection by the Reconstituted T-cell Response

**Figure 4:**
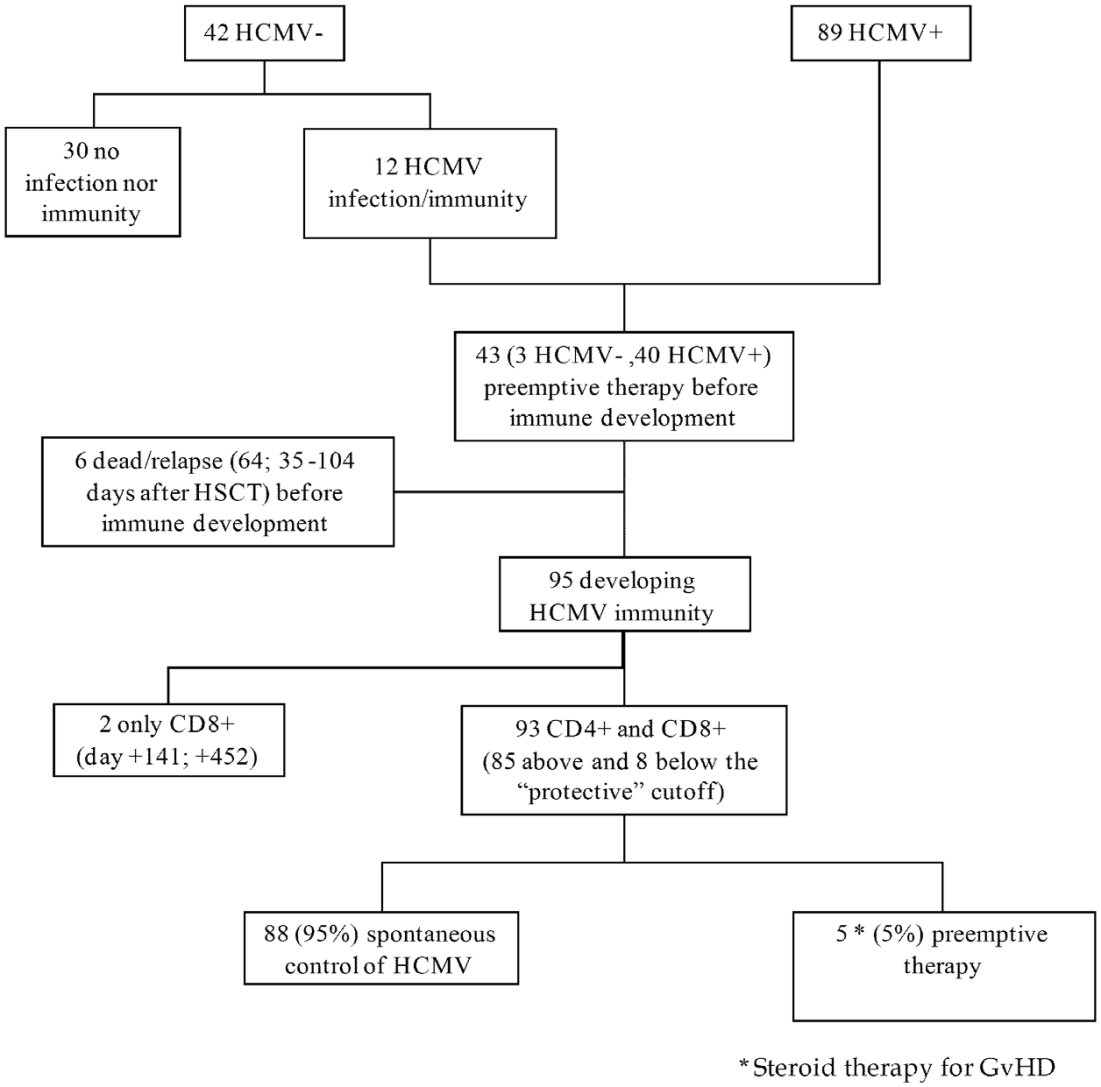
Flow-chart of HCMV-specific T-cell response. Immune control of HCMV infection in the 131 young patients enrolled in the study. During follow-up, 12/42 HCMV-seronegative and 89/89 HCMV-seropositive patients developed HCMV infection/immunity, for a total of 101 patients. Forty-three patients required pre-emptive therapy to control HCMV infection prior to development of specific immunity. Six patients died for underlying disease relapse. Of the 93 remaining patients, 88 (95%) were protected, while 5 (5%) were treated with additional courses of pre-emptive therapy because the steroid therapy employed for treating GvHD promoted reactivation of viral infection, with a viral load in blood reaching the established cutoff.

As reported in Fig. 4, of the 131 HSCT recipients enrolled in the study, 30 patients (all HCMV-seronegative not developing HCMV infection) did not have HCMV-specific T-cells after a median time of 186 (61–363) days of follow-up (none of them developed HCMV infection thereafter), and 6 patients died because of disease relapse at a median of 64 days (range 35–104) after HSCT before immune response reconstitution. Thus, 95 patients developed T-cell immunity. Of these, while 2 patients recovered CD8+ T-cells alone, 93 patients reconstituted or developed both HCMV-specific CD4^+^ and CD8^+^ T-cell numbers found to be protective in healthy subjects (>0.4 cells/µl blood) [16], and 85 of these reached protective T-cell levels previously chosen for HSCT recipients. The 8 patients reaching levels of HCMV-specific CD4^+^ and CD8^+^ T-cells below the threshold chosen for immune-compromised patients were able to control the infection without antiviral treatment.

Only 5/93 (5%) patients, after reaching the level of CD4^+^ T-cells >0.4/µl blood were given pre-emptive therapy to control HCMV infection. All these 5 patients were receiving steroids (associated with extracorporeal photochemotherapy in 3 cases, plus MMF in 1 case) for either acute or chronic GvHD for 69 (range 37–135) days, and started pre-emptive therapy upon reaching the established cut-off. In 2 of these 5 patients, loss of protective levels of CD4^+^ T-cells and IL-2 production by both CD4^+^ and CD8^+^ T-cells was observed. No immunological alterations were observed in other two patients, whereas for the remaining patient the immune response after GvHD treatment could not be determined.

In the absence of GvHD, virus-specific T-cell immune response remained stable after recovery, and no patient required anti-HCMV therapy after reconstitution or development of HCMV-specific CD4^+^ T-cells. CD8^+^ T-cells alone did not appear able to control HCMV infection. In fact, in the group of 25 patients in whom the CD8^+^ presence was observed a median time of 57 days (16–340) prior to appearance of CD4^+^ T-cells, 5 patients (20%) developed high DNAemia levels requiring antiviral treatment.

**Figure 5:**
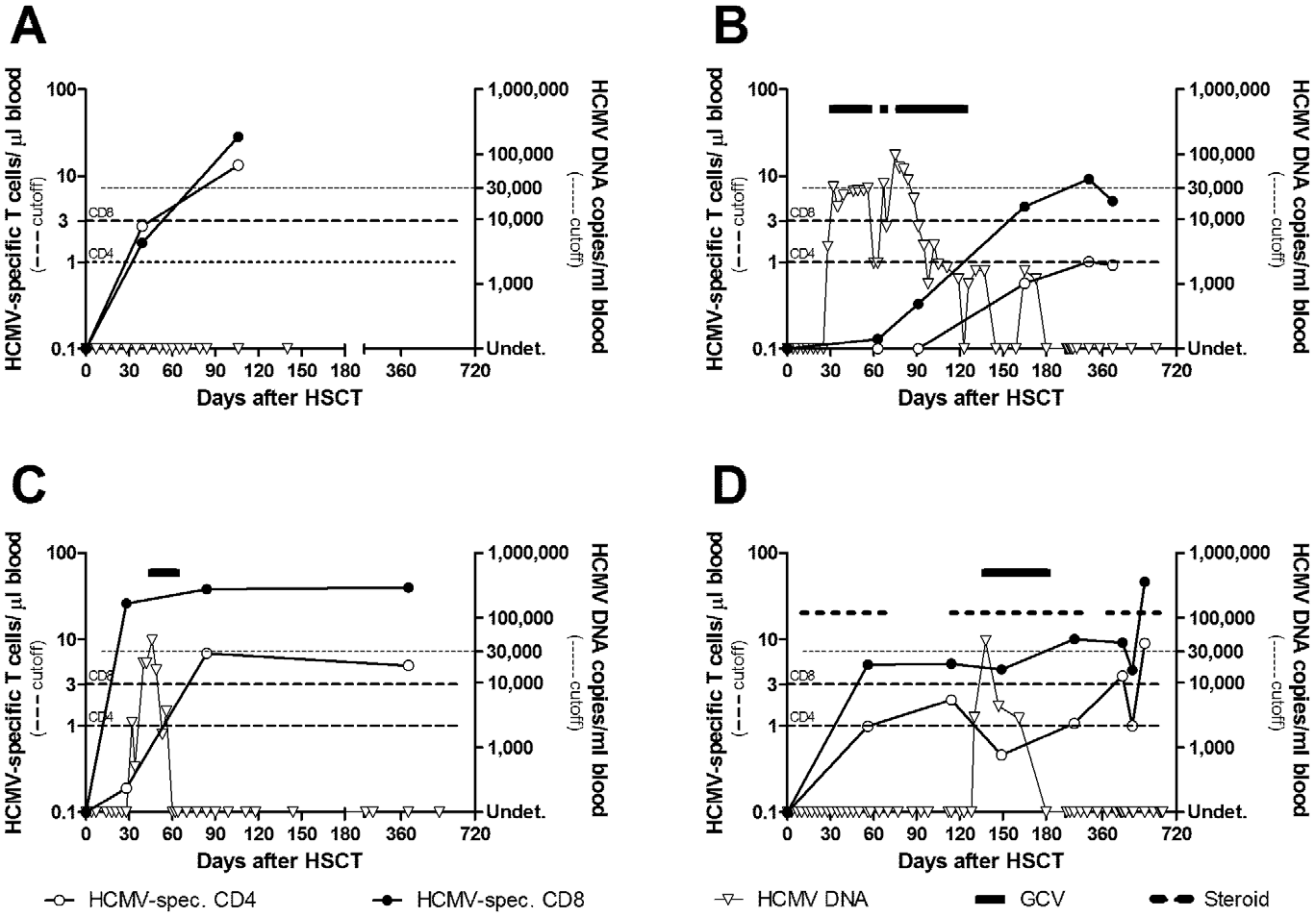
HCMV-specific T-cell response to HCMV infection in 4 young patients receiving HSCT transplantation. (A) Early specific CD4^+^and CD8^+^ T-cell response with no HCMV infection. (B) Delayed CD4^+^ and CD8^+^ T-cell response with high viral load in a patient pre-emptively treated. (C) Early CD8^+^ T-cell response which did not prevent HCMV infection until HCMV-specific CD4^+^ response appeared. (D) In the presence of acute and chronic GvHD requiring steroid treatment, specific immune reconstitution did not protect against HCMV infection, which required ganciclovir (GCV) treatment, and was eventually prevented by a protective CD4^+^ and CD8^+^ T-cell response.

Four representative cases of T-cell response to HCMV infection are described in Fig. 5.

## Discussion

This study demonstrate that: i) HCMV infection is much more frequent in seropositive than in seronegative patients; ii) as a consequence, the virus-specific T-cell response was much more frequent in HCMV-seropositive patients; iii) however, protective activity of the T-cell response against HCMV infection was detected in both seropositive and seronegative patients; iv) protection was stable and long-lasting, unless steroid therapy for GvHD was administered; v) both HCMV-specific CD4^+^ and CD8^+^ T-cells are required to confer protection against HCMV reactivation.

Although HCMV-seropositive patients showed a much higher incidence of infection than HCMV-seronegative recipients (73% *vs* 17%), EFS, OS and TRM were not different between the two groups. The finding that these parameters were similar in patient groups with high or low HCMV infection rate suggests that the impact of HCMV infection on transplant outcome is nearly abolished by the pre-emptive therapy strategy herein adopted. Support to this conclusion is provided by the observation that only one patient developed organ-specific HCMV disease (i.e. gastritis).

We used to detect the HCMV-specific immune response our recently developed methodology based on use of monocyte-derived, HCMV-infected autologous dendritic cells to stimulate T-cells [16]. This method is not HLA-restricted, takes advantage of the simultaneous expression on the DC membrane of different viral proteins, stimulates both CD4^+^ and CD8^+^ T-cells, while allowing T-cell functional evaluation. Using this method, we observed that after 9 months all HCMV-seropositive surviving patients reconstituted both CD4^+^ and CD8^+^ T-cell immunity, but one, who did not show the presence of CD4^+^ T-cells after 1-year follow-up. This means that in seropositive patients post-transplant HCMV reactivation represents the major factor driving HCMV-specific immune reconstitution. This conclusion was shared by other authors [24], [25] and the low number of cases in which immunity was reconstituted in the absence of detected infection, might be attributed to a silent infection occurring in a target organ. In seropositive donor/seronegative recipient pairs, immune reconstitution in the absence of detectable HCMV infection, might recognize similar mechanisms, although also an antigen-independent, cytokine-driven expansion of donor memory T-cells has been advocated [26].

Since more than a decade, there is a debate on whether both HCMV-specific CD4^+^ and CD8^+^ are required to confer protection against HCMV reactivation, or one of these two T-cell subpopulations is sufficient to protect from HCMV relapse. Although studies have claimed the protective role of HCMV-specific CD8^+^ cytotoxic T-cells [12], [13], results of our investigation indicate that both T-cell subsets are required for a long-lasting protection against HCMV reactivation. Conversely, some authors indicate that HCMV-specific CD4^+^ T-cells may be sufficient to predict a reliable control of HCMV infection [14], [15]. Use of immunological cut-offs, which were previously established for control of HCMV infection in pediatric patients [17], has been prospectively validated in this study conducted on a large number of patients and with a long observation time. This allowed to prove that a given level of T-cell response is able to prevent HCMV reactivation episodes and strongly suggests that immunological monitoring should be associated to virological monitoring for surveillance of HCMV infection.

We observed also that after reaching levels of both HCMV-specific CD4^+^ and CD8^+^ T cells similar to those found to be protective in immunocompetent subjects (>0,4 CD4^+^ and CD8^+^ T cells/µl blood) patients were able to control HCMV infection without need of antiviral treatment. Thus, after recovery of specific immunity, it could be possible to discontinue antiviral interventions and/or virologic monitoring, at least within 3 years after transplantation, as already proposed by others [27]. After that time, a future recommendation could include immunologic monitoring on a yearly basis. However, it is likely that the reconstituted immune system may persist lifelong, unless severe adverse events (such as disease progression or organ rejection) occur. Only in case of immune suppressive treatment for GvHD, the control of HCMV infection by the reconstituted specific T-cell pool cannot be assured, and virological monitoring should be resumed. Finally, since HCMV-specific CD4^+^ T cells were always detected in the presence also of CD8^+^ T cells, determination of virus-specific CD4^+^ T cells only, could be a good surrogate marker of complete immune reconstitution.

The immunological cut-offs of this study were calculated in the past with reference to T-cells producing IFN-γ only. However, in this study we determined also IFN-γ/IL-2 producing T-cells. In a recent report, it was shown that in HIV-infected patients, control of viral infection required the presence of both IFN-γ and IL-2 producing T-cells [28]. In this report, appearance of IFN-γ/IL-2 producing T-cells was delayed with respect to T-cells producing IFN-γ only, as already observed during development of primary immune response in the immunocompetent host [29], [30]. Thus, in the initial phase of immune reconstitution, the presence of CD4^+^ and CD8^+^ T-cells producing only IFN-γ might be sufficient to confer protection to young HSCT recipients.

In the near future, other aspects of the acquired, as well as of the innate immune response, that, so far, have only preliminarily been studied, will have to be investigated. In particular, γ/δ T cells seem to possess an important role in the protection against HCMV disease and in the resolution of HCMV infection in HSCT recipients [31], [32]. In addition, it was suggested that also CD4^+^CD25^+^ regulatory T-cells may contribute to HCMV-specific immune reconstitution [33]. Finally, natural killer (NK) cells also play a role in limiting HCMV replication [34], [35].

The immunosuppressive effect of GvHD treatment in the presence of levels of immunity above the established cut-offs, was observed in 5 patients. Two of these patients did not show levels of HCMV-specific CD4^+^ and CD8^+^ T-cells producing both IFN-γ and IL-2. Thus, this could be considered a surrogate marker of steroid-induced T-cell alteration. In previously published studies, steroid treatment was considered responsible for delayed T-cell reconstitution [36] or the presence of non-functional HCMV-specific T-cells [37].

The only factors predicting delay in immune reconstitution were T-cell depletion of the graft and use of cord blood cells. We already reported that graft T-cell depletion is associated with delay in HCMV-specific T-cell reconstitution [21]. Cord blood T cells are immunologically naïve, this preventing the possibility that the recipient could benefit from the adoptive transfer of pathogen-specific immunity. Moreover, as CBT recipients are given steroids for GvHD prophylaxis during the first 30 days after HSCT, one cannot exclude that steroids could contribute to the delayed HCMV-specific immune reconstitution. The lack of impact of donor serostatus on HCMV-specific immune reconstitution in children receiving HSCT (in contrast to what observed in adult patients receiving HSCT from a seronegative donor in whom a delayed immune reconstitution occurs [38]), confirms our previous observation made in a smaller cohort of pediatric patients [17]. It is possible that in children the presence of a better thymic function facilitates the development of a primary immune response in the absence of HCMV-specific memory T cells in the graft.

In summary, we demonstrated that in young HSCT recipients monitoring of HCMV-specific immune recovery can usefully complement virological monitoring for deciding which patients need antiviral treatment.
